# Role of Surgery in Breast Metastasis from Carcinoma of the Cervix

**DOI:** 10.4103/0973-1075.78454

**Published:** 2011

**Authors:** Parveen Yadav, NML Manjunath, SVS Deo, NK Shukla, Prashant Durgapal, Dillip K Muduly

**Affiliations:** Department of Surgical Oncology, BRAIRCH, New Delhi, India; 1 Department of Pathology, AIIMS, New Delhi, India

**Keywords:** Cervix, Metastasis, Palliative

## Abstract

Carcinoma of the cervix is the most common malignancy among women in India. Although metastatic disease is common, metastasis to breast is rare. A limited number of case reports are published in the world literature. Most of the previous reports of metastatic cervical carcinoma to breast are either autopsy series or widely disseminated disease where no treatment options were available. A rare case of cervical carcinoma presenting as metastasis in breast is reported here where palliative mastectomy improved the general condition of the patient. A female patient aged 58 years was diagnosed and treated for cervical carcinoma, FIGO stage 2B. Four months after the treatment which included both external beam and intracavitory radiotherapy, the patient presented with breast and lung metastasis. Palliative mastectomy was done which improved the general condition of the patient. Metastatic carcinoma of the cervix can present as a case of breast carcinoma. In an appropriate setting, this possibility should be kept in mind. Palliative mastectomy should be offered for patients of cervical carcinoma with metastasis to breast when needed.

## INTRODUCTION

Metastasis to breast from extramammary site is uncommon. In clinical and autopsy studies an incidence of 0.5% to 6.6% have been reported. Most of the metastasis to breast are from malignant melanoma, Lymphoma, Carcinoma lung, stomach and ovary but metastasis from primary cervical cancer is rare. A case of primary cervical carcinoma with breast metastasis is reported here.

## CASE REPORT

**Figure 1:**
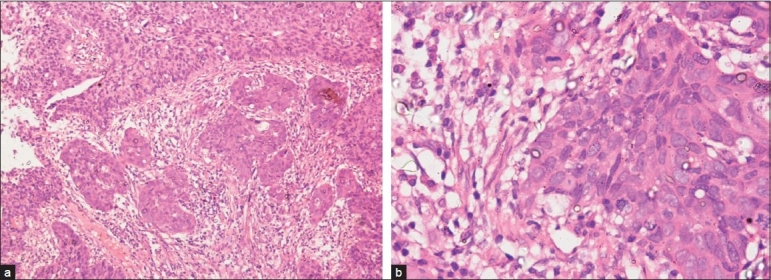
(a) Histopathological examination of punch biopsy specimen of cervical carcinoma, ×10 low power showing large cell nonkeratinizing squamous cell carcinoma. (b) Histopathological examination of punch biopsy specimen of cervical carcinoma, ×40 high power showing large cell nonkeratinizing squamous cell carcinoma

A female patient aged 58 years presented to a tertiary cancer care unit with a history of bleeding from the vagina and low back ache since 4 months. On clinical evaluation, patient’s general condition was good, and per vaginal examination revealed a cervical growth with left parametrial involvement not up to the pelvic wall. Biopsy of the cervical growth showed a nonkeratinizing squamous cell carcinoma [Figure 1]. Her cystosigmoidoscopy was normal. Final diagnosis was carcinoma of the cervix, FIGO – International Federation of Gynecology and Obstetrics – stage 2B. The patient received external beam radiotherapy 50 Gy/27 fractions followed by high dose rate intracavitary brachytherapy (3 sessions each of 7 Gy) from October 31, 2008, to January 23, 2009. The patient was cured on clinical and radiological evaluation and was on regular follow-up.

Four months after the treatment, on follow-up evaluation the patient gave a history of lump in the left breast. On examination, a lump measuring 8 × 7 cm was palpable in the upper outer quadrant of the left breast with no evidence of skin or chest wall invasion. Mammographically, a high-density mass with ill-defined margins was noted in the upper outer quadrant; no calcification was noted. On ultrasonographic correlation, the mass was hypoechic with internal vascularity. Tru-Cut biopsy of the lump was done and histopathological examination showed features suggestive of an infiltrating ductal carcinoma with negative estrogen and progesterone receptors. Her primary site was clinically evaluated and was normal. A diagnosis of breast carcinoma as second primary was made and the evaluation was initiated on the same lines.

As a part of metastatic work-up and to know the status of the primary carcinoma, a positron emission tomographic scan was done which showed uptake in the left breast and left lung. Her bone scan and ultrasonography (USG) of the abdomen were normal. Chemotherapy for metastatic breast carcinoma was planned. The patient received two cycles of 5-flurouracil, epirubicin, and cyclophosphamide with poor tolerance. Meanwhile her breast mass progressed to 20 cm in size and was ulcerated.

**Figure 2:**
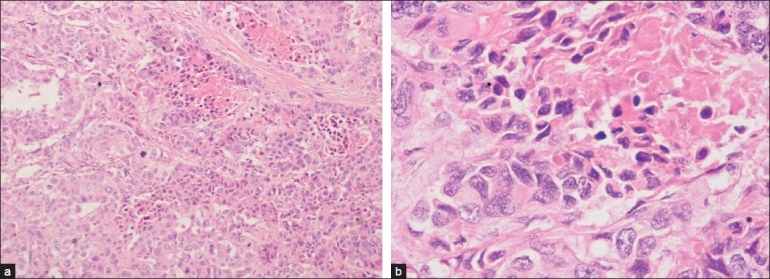
(a) Histopathological examination of mastectomy specimen, ×10 low power showing squamous cell carcinoma with intraepithelial keratin. (b) Histopathological examination of mastectomy specimen, ×40 high power showing squamous cell carcinoma with intraepithelial keratin

In view of the poor tolerance to chemotherapy and the progressive fungating mass lesion of the breast, palliative mastectomy was done and specimen sent for histopathological examination. As a histological surprise, metastatic squamous cell carcinoma consistent with carcinoma of the cervix was reported [Figure 2]. Retrospective correlation of slides revealed an adenosquamous type of cervical carcinoma with metastasis to breast.

After palliative mastectomy, patient’s general condition improved and on the last follow-up, both primary local and mastectomy sites were clinically normal. Biopsy of lung lesions was done which again revealed metastatic carcinoma of the cervix. Palliative chemotherapy for metastatic cervical carcinoma is being planned. At the time of reporting this case, patient was alive and with disease in the left lung.

## DISCUSSION

Carcinoma of the cervix is the commonest malignancy among females in India. The natural history of cervical carcinoma is of a systematic pattern of metastatic progression, initially to primary echelon nodes followed by para-aortic and then distant sites. The most frequent sites of distant recurrence are lungs, extra pelvic nodes, liver, and bones.[1]

The autopsy incidence of metastasis to breast with extramammary primaries ranged from 1.4% to 6.1%,[2,3] whereas clinically observed rates are 1.2%.[4] Melanoma is the most frequent tumor metastatic to breast.[2,5] Other common malignancies which metastasize to breast are carcinoma from the opposite breast, lymphomas, and epithelial carcinomas notably bronchogenic carcinoma.[4,6] Reported cases of gynecological malignancy metastasizing to breast mostly involve ovarian cancer.[7] Metastasis from endometrial, vulval, and cervical carcinoma have also been reported although very rarely.

The first case of cervical carcinoma metastatic to breast was reported by Spreet and Greeley.[8,9] From then onward, few reports have been published in the world literature.[3,8,10,11] Most of the previous reports of metastatic cervical carcinoma to breast are either autopsy series or widely disseminated disease where no treatment options were available.

A rare case of cervical carcinoma presenting as metastasis in the breast is reported here. On retrospective review of this rare clinical situation, it was confirmed that breast metastasis from cervical carcinoma has some characteristic features. It presents in the upper outer quadrant. Skin and chest wall invasion is late and it presents as a discrete mobile swelling in a subcutaneous location.[4,12,13] Mammographically, these tumors show well-defined or irregular soft tissue densities without calcification.[14] In terms of USG, they show low-level internal echoes in a well-defined mass and no distal acoustic enhancement.[10,14] All these features correlated well with our case.

The diagnostic dilemma in our case was the pathological report of infiltrating ductal carcinoma in the Tru-Cut biopsy of the breast lesion. In the literature, similar cases have been reported.[11,12] In these cases also, as confirmed in our case the primary lesion was adenosquamous type of cervical carcinoma. This can be explained by the fact that the Tru-Cut biopsy represents only part of the pathological tissue. In an appropriate clinical setting where the metastatic lesions are strongly suspected, thorough evaluation and correlation of biopsy specimens avoids dilemma and initiation of appropriate management at the earliest.

The management of cervical carcinoma with metastasis to the breast is not clear as it is a rare clinical entity. In the literature, majority of the reports contemplate only palliative chemotherapy and radiotherapy. Only one case of palliative mastectomy for cervical carcinoma with breast metastasis is reported.[11] Palliative mastectomy needs to be considered as treatment for bleeding and fungating tumors as in our case.

Mastectomy improved patient’s general condition. The aim of palliative care is physical, psychological, and social improvement which was appreciated in our patient. The lesson learnt is that metastatic cervical carcinoma can present as a case of carcinoma of the breast. In an appropriate setting, this possibility should be kept in mind. Palliative mastectomy should be offered for patients of cervical carcinoma with metastasis to the breast.
